# SP600125 enhances C-2-induced cell death by the switch from autophagy to apoptosis in bladder cancer cells

**DOI:** 10.1186/s13046-019-1467-6

**Published:** 2019-11-04

**Authors:** Haiyang Yu, Chun-Li Wu, Xiangyu Wang, Qianhong Ban, Chunhua Quan, Mengbo Liu, Hangqi Dong, Jinfeng Li, Gi-Young Kim, Yung Hyun Choi, Zhenya Wang, Cheng-Yun Jin

**Affiliations:** 1 Tianjin State Key Laboratory of Modern Chinese Medicine, Tianjin University of Traditional Chinese Medicine, 312 Anshanxi Road, Nankai District, Tianjin, 300193 China; 2 School of Pharmaceutical Sciences, Key Laboratory of State Ministry of Education, Key Laboratory of Henan province for Drug Quality Control and Evaluation, Collaborative Innovation Center of New Drug Research and Safety Evaluation, Zhengzhou University, 100 Kexue Avenue, Zhengzhou, 450001 Henan China; 3 Kidney Transplantation, The First Affiliated Hospital of Zhengzhou University, No. 1 Jianshe Road, Erqi District, Zhengzhou, 450001 Henan China; 4 Department of Marine Life Sciences, Jeju National University, Jeju, 63243 Republic of Korea; 5 Department of Biochemistry, College of Oriental Medicine, Dong-Eui University, Busan, 47227 Republic of Korea

**Keywords:** SP600125, Autophagy, Apoptosis, Bladder cancer, C-2, SQSTM1/p62

## Abstract

**Background:**

A natural compound Jaspine B and its derivative possess potential anti-cancer activities; However, little is known about the underlying mechanism. Here, the role of a new autophagy inducer Jaspine B derivative C-2 in suppressing bladder cancer cells was researched in vitro and in vivo.

**Methods:**

The underlying mechanisms and anticancer effect of C-2 in bladder cancer cells were investigated by MTT, western blotting, immunoprecipitation and immunofluorescence assays. The key signaling components were investigated by using pharmacological inhibitors or specific siRNAs. In vivo, we designed a C-2 and SP600125 combination experiment to verify the effectiveness of compound.

**Results:**

C-2 exhibits cytotoxic effect on bladder cancer cells, and JNK activated by C-2 triggers autophagy and up-regulates SQSTM1/p62 proteins, contributing to activation of Nrf2 pathway. Utilization of JNK inhibitor SP600125 or knockdown of JNK by siRNA potentiate the cytotoxicity of C-2 through down-regulation of p62 and LC3II proteins and up-regulation of active-Caspase3 proteins, enhance the cell death effect, facilitating the switch from autophagy to apoptosis. In vivo study, C-2 suppresses tumor growth in a xenograft mouse model of EJ cells without observed toxicity. Combined treatment with SP600125 further enhances tumor inhibition of C-2 associated with enhanced activation of caspase3 and reduction of autophagy.

**Conclusions:**

It reveals a series of molecular mechanisms about SP600125 potentiate the cytotoxicity and tumor inhibition of C-2 in bladder cancer cells through promoting C-2-induced apoptosis, expecting it provides research basis and theoretical support for new drugs development.

## Background

**Fig. 1:**
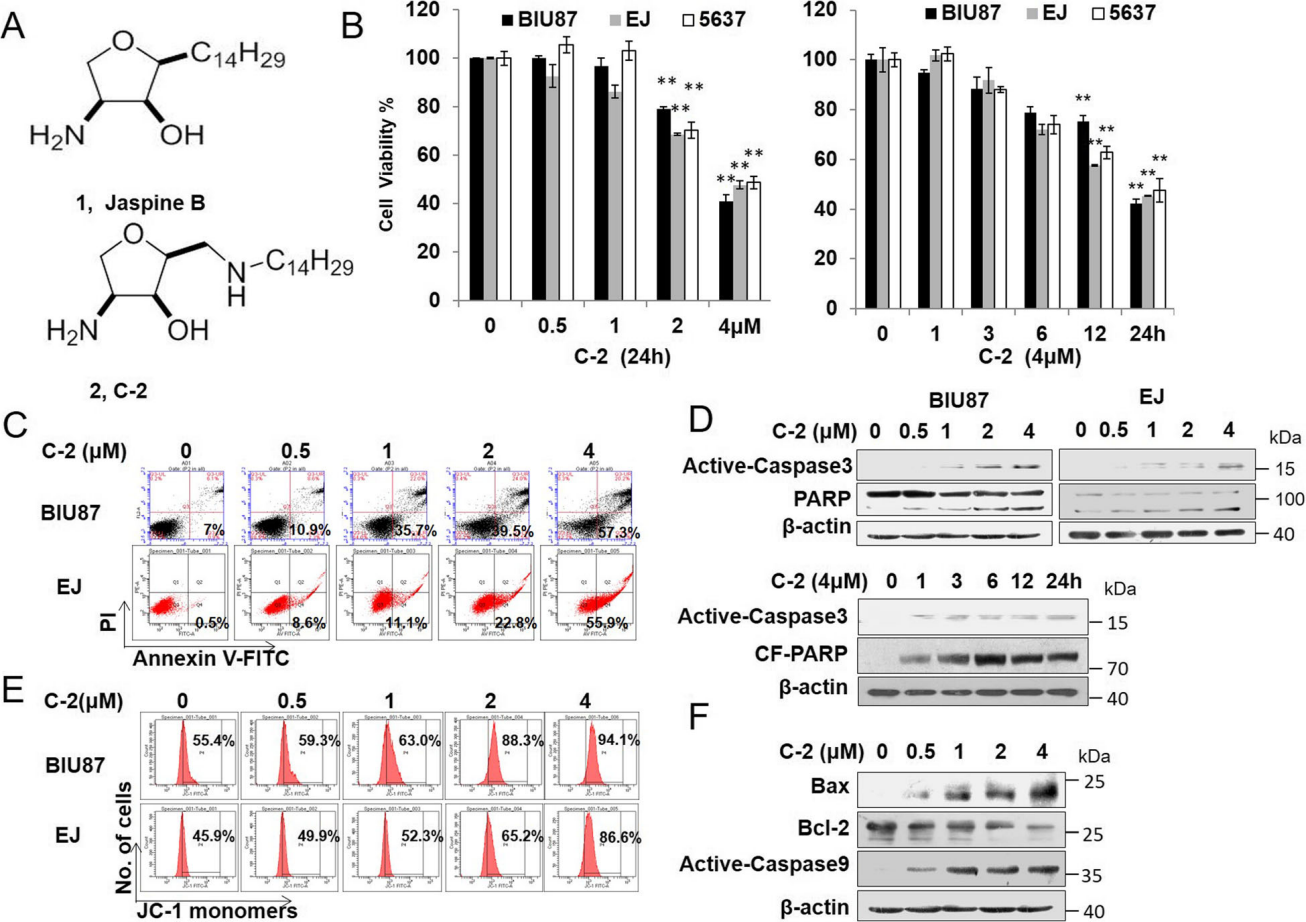
C-2 significantly reduced the viabilities of human bladder cancer cells and induced apoptosis associated with the mitochondrial pathway. **a** structure of Jaspine B and C-2. **b** The effect of C-2 in reducing cell viabilities of bladder cancer cells (BIU87, EJ and 5637) measured by MTT assay. Cells were treated with the indicated concentrations of C-2 for 24 h and 4 μM of C-2 at indicated time points. **P < 0.02 vs. control group. **c** A dose-dependent induction of apoptosis by C-2 was demonstrated through flow cytometric analysis of Annexin V/PI stain assay. **d** Active-casepase 3, PARP and CF-PARP proteins expression were detected by western blotting at a dose- and time- dependent manner in BIU87 cells. **e** The mitochondria membrane potential (ΔΨ) was decreased by C-2 treatment. BIU87 cells were treated with vehicle control or varying concentrations of C-2 for 24 h. Membrane potential was measured by JC-1 dye retention using Flow Cytometry. **f** The protein levels of Bax, Bcl-2, XIAP and Active-caspase9 were determined by western blotting assay in BIU87 cells at indicated concentrations for 24 h

**Fig. 2:**
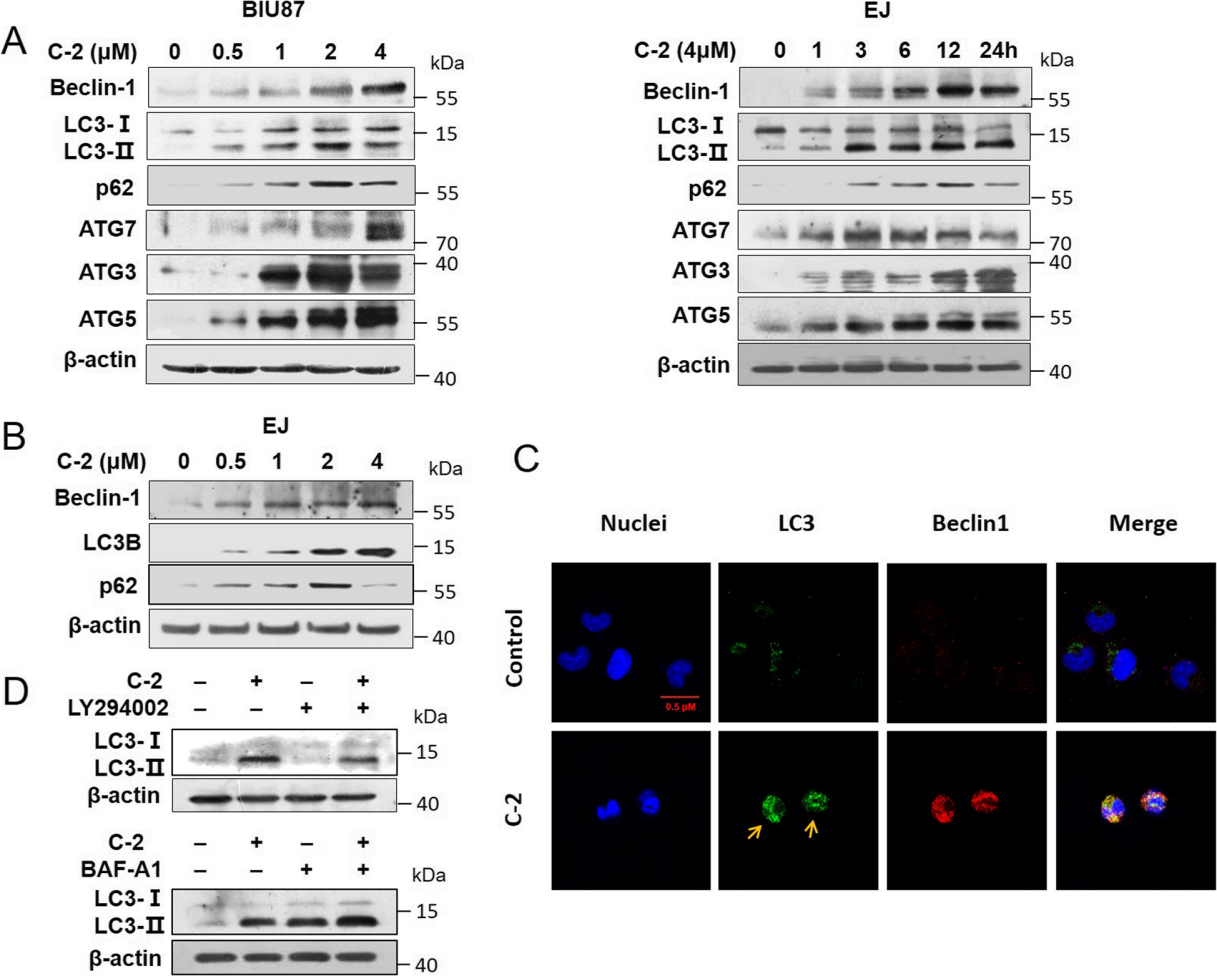
C-2 induced autophagy of bladder cancer cells. **a** The expression levels of C-2-induced autophagy related protein Beclin-1, LC3, p62, ATG3, ATG5 and ATG7 were detected by western blotting in BIU87 and EJ cells at the indicated concentrations or treated with 4 μM of C-2 at the indicated time points. **b** The expression levels of C-2-induced autophagy related protein Beclin-1, p62 and LC3 were detected by western blotting in EJ cells at the indicated concentrations. **c** BIU87 cells were treated with 4 μM of C-2 for 12 h and 24 h. The treated and untreated samples are stained with LC3 antibody (Green) and Beclin1 antibody (Red) and DAPI (Blue) (magnification, 600X). **d** BIU87 cells were pretreated with LY294002 (25 μM) and Baf-A1 (0.5 μM) for 1 h and then treated with 4 μM of C-2 for an additional 24 h. Western blotting was performed to determine the effect of LY294002 and Baf-A1 on the expression of LC3-II in BIU87 cells

Bladder cancer is a common urologic cancer and is associated with substantial morbidity, mortality and cost [1]. Jaspine B, a novel anhydrophytosphingosine, was first isolated from a marine sponge *Pachastrissa sp.* in 2002 [2] (Fig. 1a), which exhibited a potent cytotoxicity at an IC_50_ level of 0.01 μg/mL against several tumor cell lines. Our previous study reported that a new series of Jaspine B derivatives were designed and synthesized, among them, compound 7f was discovered as an autophagy inducer is associated with the up-regulation of LC3 and Beclin-1, and showed the best overall cytotoxicity on PC-3 cells [3]. And in that article, another compound 7 g (Fig. 1a, Fig. 2) also has significant cytotoxicity and could induce cell autophagy, due to the efficiency of Jaspine B derivatives was investigated in bladder cancer cells rarely, and the specific autophagy effect of compound 7f in PC3 cells had not been investigated deeply. Therefore, compound 7 g was selected and given chemical name of C-2 to further research autophagy mechanism and its effect on bladder cancer cells and to evaluate its antitumor activities in this study.

Programmed cell death is categorized into two types: apoptosis (type I) and autophagy (type II). Autophagy is a cellular degradation mechanism while apoptosis is a self-killing mechanism [4]. Both processes can be triggered after cell injury and play an essential role in the development and tissue homeostasis of multicellular organisms, and the activation of apoptotic pathway are critical in suppressing the survival of different cancer cells [5]. Moreover, the anti-apoptotic proteins of the Bcl-2 family such as Bcl-2 and Bcl-xL could bind Beclin-1 to inhibit autophagy. The effect of autophagy in cancer cells’ response to chemotherapy is also complex: acting as a tumor suppressor and as a promoter of cell survival by increasing tumor growth [6].

JNK is a c-Jun N-terminal kinase and also known as a stress-responsive protein kinase of the MAPK family. JNK is primarily activated by various environmental stress include several antitumor proteins, stress signals and chemotherapy drugs, and JNK plays a critical role in the regulation of cell growth, differentiation, apoptosis and other signaling pathways [7]. Furthermore, growing evidence in recent years demonstrates JNK also contributes to autophagic induction through phosphorylating Bcl-2 and Bcl-xL to active Beclin-1 in response to various stress signals [8].

SQSTM1 (sequestosome 1)/p62 is an autophagy adaptor protein, binding ubiquitylated protein aggregates and delivering them to the autophagosomes. Recently, p62 has emerged as a multifaceted adaptor protein that exhibits diverse biological functions through the interaction with numerous proteins, and p62 is also identified as a new metabolic tumor suppressor [9]. The Kelch-like ECH-associated protein 1 (Keap1) and nuclear factor erythroid 2-related factor 2 (Nrf2) pathway is essential for cytoprotection against oxidative stress [10]. p62 could interacts with the Nrf2-binding site on Keap1, resulting in stabilization of Nrf2 and transcriptional activation of Nrf2 target genes, then perform cellular defense mechanisms against oxidative stresses [11]. Therefore, both the autophagy and Keap1-Nrf2 pathways are intimately linked by p62, antagonizing cellular stress by up-regulating a battery of antioxidant and cellular defense genes. The detailed mechanistic understanding of the interaction between the Keap1-Nrf2 and autophagy is a clear demonstration of the ongoing need for detailed mechanistic pictures of biological and pathological phenomena to facilitate the discovery of new therapies [12].

In many stress pathways, autophagy and apoptosis seem to co-exist simultaneously or interacted with the aid of mutual proteins, serving as a switching points critical to the ultimate outcome of the cells [13]. In particular situations, autophagy may antagonize apoptosis, and the cytoprotective function of autophagy in cancer cells has been suggested as a potential mechanism for chemoresistance [14]. However, when the intensity of the stress overcomes the protective barrier of autophagy, the cell becomes rapidly death. Therefore, the switch from autophagy to apoptosis suggested that autophagy induction was responsible for apoptotic lag phase and was an adaptive response [15]. The role of autophagy in cancer cells is controversial and it is urgent to research the effect of that on cancer.

Herein, in our study, C-2 exhibited cytotoxic effect against bladder cancer cells. We discussed the mechanism of C-2 on bladder cancer cells BIU87 and EJ with apoptosis and autophagy. Destruction of the crosstalk between p62 and Keap1-Nrf2 pathway through inhibiting the initiation of JNK-mediated autophagy by SP600125 triggering the switch from autophagy to apoptosis, which could be utilized for sensitizing bladder cancer cells to treatment with C-2. We also evaluated antitumor activity of C-2 or combine with SP600125 in EJ tumor bearing xenograft mice model.

## Methods

### Reagents

Fetal bovine serum (FBS), RPMI-1640, and penicillin-streptomycin were purchased from HyClone (Victoria, Australia). 3-(4,5-dimethyl-thiazol-2-yl)-2, 5-diphenyltetra-zolium bromide (MTT) and JC-1 fluorescent dye (Sigma-Aldrich, St Louis, MO), LY294002 and SP600125 (selleck, Texas, Houston), Protein A/G plus Agarose immunoprecipitation reagent (Santa Cruz, CA), DAPI (C0060, Solarbio, Beijing, China), lysis buffer (Beyotime, Shanghai, China), Annexin- V/FITC Apoptosis Detection Kit (BestBio, Shanghai, China). Antibodies specific for β-actin (sc-1615), Bax (sc-493), Bcl-2 (sc-783), Nrf2 (sc-13,032), c-Jun (sc-74,543) and p-c-Jun (sc-53,182) were obtained from Santa Cruz Biotechnology (Santa Cruz, CA). p-Nrf2 (ab76026), p62 (ab56416), Anti-active caspase 3 (ab2302) and anti-active caspase 9 (ab2324) were purchased from Abcam (Cambridge, MA). SAPK/JNK siRNA (#6232), p62 siRNA (#6394), Poly (ADP-ribose) polymerase-1 (PARP-1) (#9532), Cleaved PARP (#5625), NQO1 (#3187), Keap1 (#4617), Bcl-xL (#2764), XIAP (#14334), JNK (#9252), p-JNK (#9255), LC3B (#3868S) and Autophagy antibody sampler kit (#4445) including Beclin-1, ATG16, ATG3, ATG7 and ATG5 were purchased from Cell Signaling Technology (Danvers, MA). The enhanced chemiluminescence (ECL) kit was purchased from ThermoFisher (Waltham, MA).

### Cell culture and proliferation

BIU87, 5637 and EJ (human Bladder cancer cell) were obtained from ATCC. The used cells were resuscitated within 1 month, and cells preserved in liquid nitrogen and were expanded at low passages. Cells were in the logarithmic phase of growth for all experiments. Cells were cultured at 37 °C in an atmosphere containing 5% CO_2_ with RPMI 1640 medium supplemented and 10% heat-inactivated fetal bovine serum, 100 U/ml penicillin and 0.1 mg/ml streptomycin. Cells were seeded into a 96-well plate at a density of 3,000 (100 μl) cells per well for 24 h and followed by compound added (200 μl) to the respective well in the indicated concentrations. Next, 20 μl of 5 mg/ml MTT per well was added to the medium, and the cells were incubated for 4 h at 37 °C and 5% CO_2_. After removing the culture medium, 150 μl of DMSO was added to dissolve the formazan crystal. The absorbance was read by enzyme labeling instrument with 490 nm wavelength measurement. The viability of the untreated cells was set as 100%, and the viability of the other groups was calculated by comparing the optical density reading with the control. The IC_50_ value was calculated using nonlinear regression analysis.

### Apoptosis analysis

BIU87 and EJ Cells were seeded at 1 × 10^5^ cells per well in a 6-well plate and cultured for 24 h. Next, the cells were exposed to different concentration of C-2 for 24 h. After that, the cells were collected and washed with PBS twice, incubated with fluorescein isothiocyanate (FITC)-conjugated Annexin V and PI by following FITC Annexin V/PI apoptosis kit instruction. Apoptotic cells were detected by flow cytometer. Annexin V+/PI- staining cells were counted as early apoptosis while Annexin V+/PI+ positive staining cells as late apoptotic/necrotic cells.

### Measurement of loss of mitochondrial membrane potential (MMP, ΔΨ)

Cells were seeded at 1 × 10^5^ per well in 10% FBS RPMI-1640 into a 6-well plate, and treated with indicated concentration of C-2 for 24 h. Then JC-1 (2.5 μg/ml) probe for measurement of MMP, were added and incubated with an equal volume of cell suspension at 37 °C for 10 min and rinsed twice with PBS. The concentration of retained JC-1 dye was determined by a flow cytometer (BD Biosciences).

### Western blot

Cells (6 × 10^5^) were cultured in each 100-mm plate and treated with the indicated concentration of C-2 for 24 h. Cells then were collected and lysed with ice-cold lysis buffer (Beyotime, Shanghai, China). After centrifugation at 12,000 rpm/min for 30 min, protein concentrations of the lysates were determined by the micro-BCA protein assay kit. The total cellular protein extracts were boiled with 5 × loading buffer, separated by SDS-PAGE and transferred to nitrocellulose membrane. After blocking with 5% skimmed milk in PBST for 2 h, the membranes were incubated with appropriate antibodies overnight at 4 °C, followed by HRP conjugated anti-mouse, anti-goat or anti-rabbit secondary antibodies. The detection of specific proteins was carried out with an ECL Western blotting kit according to the recommended procedure.

### Immunoprecipitation

Cells were cultured in 100-mm dishes, treated as indicated in figure legends, after treatments, cells were collected and lysed with ice-cold lysis buffer (Beyotime, Shanghai, China). Briefly, 500 μg cell lysates were coupled to 1 μg Beclin-1 antibody in lysis buffer for 30 min at 4 °C. Then, 20 μl of protein A/G Agarose beads (50%) were added by rotating overnight at 4 °C. The beads were washed five times with PBS, then the samples were boiled for 5 min with 40 μl of 1 × loading buffer, and separated by 12% SDS-PAGE gel. The protein levels of Bcl-xL, Bcl-2 and Beclin-1 were measured by Western blot analysis.

### Immunofluorescence analysis of LC3 and Nrf2

BIU87 cells were treated with 4 μM of C-2 for 12 h. Cells were fixed with 4% paraformaldehyde in PBS for 30 min, permeabilized with 0.1% Triton X-100, and blocked with 10% normal goat serum for 30 min. Incubation with primary antibodies against LC3 and Nrf2 was done overnight at 4 °C. After washing, cells were exposed to FITC-conjugated antibody (goat-anti-rabbit Ig (H + L) -FITC). After washing, the nuclei were visualized with 2 μg/ml DAPI solution (dissolved with PBS) and added 10 min before imaging. The confocal microscopy (A1R+, Nikon, Tokyo, Japan) was used for co-localization analysis.

### Knockdown of JNK and p62 by siRNA

The complex of Short-interfering RNAs for JNK and p62 with lipofectamine 2000 were prepared, BIU-87 cells were seeded in a 6-well plate the day before transfection at 30% confluency. 36 h after transfection, 4 μM of C-2 was added to the culture for 12 h and MTT analysis was determined to examine C-2-induced cytotoxicity, and knockdown confirmed by Western blot.

### Immunohistochemistry (IHC)

Paraffin-embedded tissue sections were dewaxed and rehydrated, washed by PBS, following the antigen retrieval, endogenous peroxidase were blocked by 3% H_2_O_2_ for 20 min, and normal goat serum was used to block non-specific binding sites for 20 min, the sections were then incubated with active-caspase3, p62 and LC3 antibody diluted 1:50 in PBS at 4 °C overnight. Peroxidase-conjugated anti-rabbit antibodies were used for secondary detection, the reaction was revealed with diaminobenzidine (DAB). Sections were counterstained with hematoxylin.

### Tumor xenograft growth assay in vivo

Animals were treated according to protocols established by the ethics committee of Zhengzhou University and the experiments in vivo were conducted according to the approved guidelines and approved by the ethics committee of Zhengzhou University. Forty male nude mice (5 weeks-old) were purchased from the Chinese Academy of Sciences (Beijing, China). Cells were digested and resuspended with PBS at a density of 1 × 107 cells/ml. Cell suspension (200 μl) was subcutaneously injected into the nude mice on the backside. Once tumors volume reached about 100 mm^3^ in size, mice were randomly divided into 4 groups (*n* = 5 per group). Group I received saline vehicle only and served as control. Group II received 10 mg/kg C-2 by intraperitoneal injection (i.p) every day. Group III received 5 mg/kg SP600125 (i.p) every day whereas Group IV received 10 mg/kg C-2 as well as 5 mg/kg SP600125 (C-2/SP600125) every day till day 21. Mice were sacrificed at day 21 due to tumor burden. Tumor growth was monitored by tumor volume which was measured with calipers and calculated according to the formula, V = 0.5 × (length × width^2^). Finally, tumors were harvested after 21 days (21 injections), body weight, tumor volume and tumor weight were measured.

### Statistical analysis

The data are expressed as means ± SD. Significant differences between the groups were determined using the unpaired Student’s t-test. The statistically variation *P* < 0.05 deemed as statistically significant.

## Results

### C-2 significantly reduced the viabilities of human bladder cancer cells and induced apoptosis associated with the mitochondrial pathway

To evaluate the effects of C-2 on human bladder cancer cells, three bladder cancer cell lines (BIU87, EJ and 5637) were incubated with C-2 for indicated time points and concentrations, and then the effects of C-2 on reducing cell viabilities were measured by an MTT assay. As shown in Fig. 1b, following treatment with C-2, the viability of the bladder cancer cells decreased in a dose- and time-dependent manner with IC_50_ values of 3.315, 3.487, 3.724 μM respectively, and BIU87 and EJ cells showed the most sensitivity to C-2 treatment. Taken together, these results suggested that C-2 has cytotoxicity against bladder cancer cells.

Further experiments were conducted to determine whether the inhibition of C-2 on the viability of bladder cancer cells was the result of apoptotic cell death. Flow cytometry analysis and western blot results showed that C-2 dramatically increased the early and late apoptotic cells as well as the expression levels of Active-Casepase3 and CF-PARP proteins in BIU87 and EJ cells after 24 h treatment compare with the control group (Fig. 1c, d and Additional file 1: Figure S1A, B). Next, in order to confirm the involvement of mitochondrion in the induction of apoptosis caused by C-2, the mitochondrial fluorescent probe JC-1 was used to measure mitochondrial membrane potential. After treatment with C-2, a right shift peak can be observed obviously compare with control (Fig. 1e), indicating that mitochondrial depolarization was induced by C-2 in BIU87 and EJ cells. Western blot results showed that increasing levels of Bax and Active-caspase9 as well as decreasing expression levels of Bcl-2 proteins in dose-dependent manner in BIU87 cells (Fig. 1f and Additional file 1: Figure S1C). These results together indicated that the mitochondrial pathway involved in C-2 induced apoptosis in bladder cancer cells.

### C-2 induced autophagy of bladder cancer cells

As previously reported, the synthetic C-2 homologous series of Jaspine B derivatives compound 7f resulted in autophagy in PC3 cells [3], therefore, we examined whether C-2 could trigger autophagy in bladder cancer cells in this study. BIU87 cells were treated with C-2 in dose- or time-dependent manners, results showed that the expression level of LC3-II, Beclin-1, p62, Atg7, Atg3 and Atg5 proteins increased (Fig. 2a). Similarly, the expression level of LC3-II, p62 and Beclin-1 proteins also increased in EJ cells treated with C-2 in dose-dependent manners (Fig. 2b). Immunoflorescence assay showed that punctate LC3 and Beclin1 as represented by green and red staining in BIU87 cells significantly enhanced after treated with 4 μM of C-2 for 12 h and 24 h (Fig. 2c). In addition, we evaluated the status of autophagic flux with an early stage autophagy inhibitor LY294002 that inhibits formation of autophagosomes and a late stage autophagy inhibitor Baf-A1 that blocks fusion with lysosomes [16]. LY294002 reduced the expression of LC3-II in C-2 treated BIU87 cells, while Baf-A1 caused an additive expression of LC3-II (Fig. 2d). Overall, C-2 induced the autophagy flux.

### C-2-induced autophagy is associated with JNK pathway by alleviating the suppression of Bcl-2 and Bcl-xL on Beclin-1 in bladder cancer cells

**Fig. 3:**
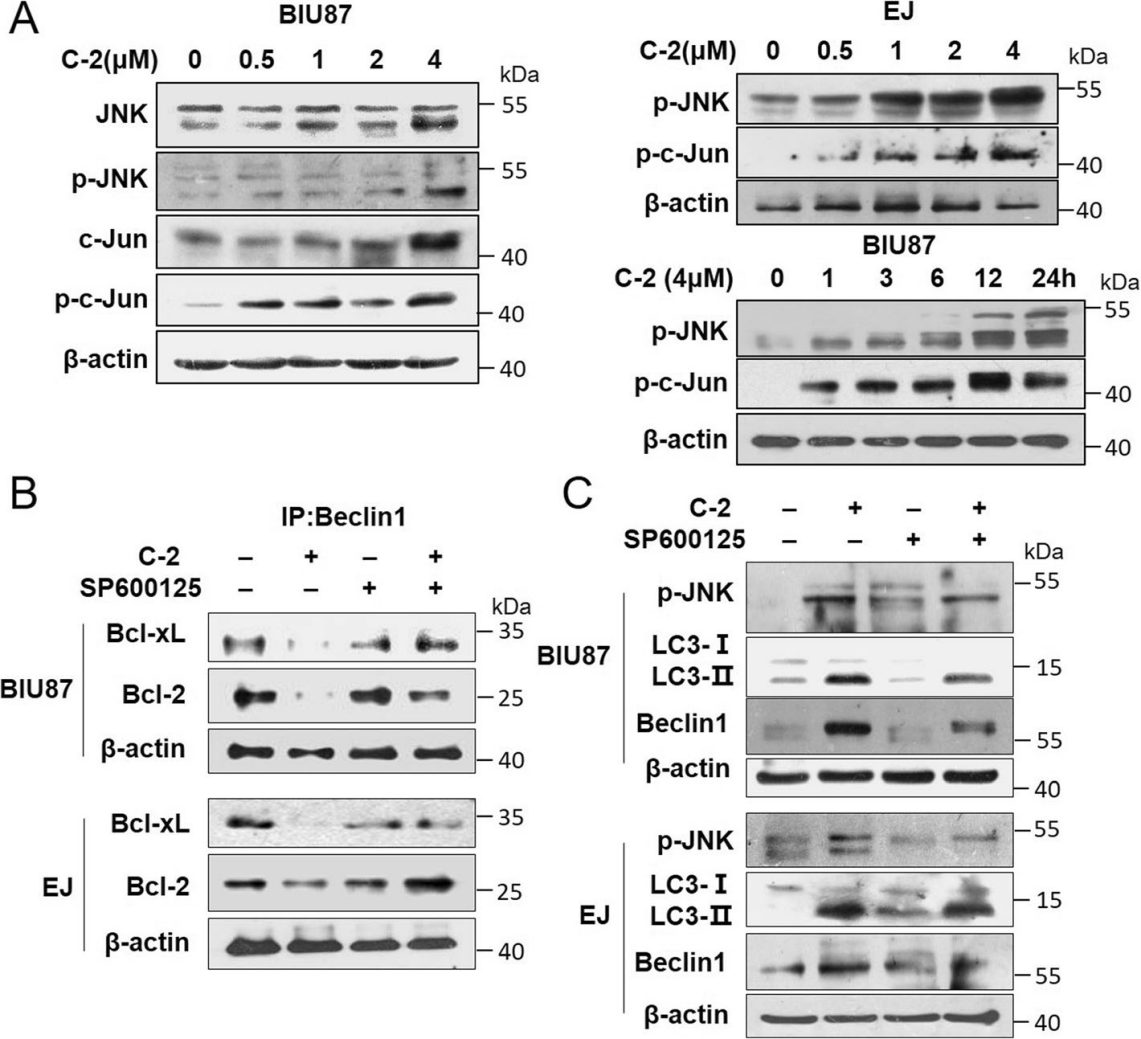
C-2-induced autophagy is associated with JNK pathway by alleviating the suppression of Bcl-2 and Bcl-xL on Beclin-1 in bladder cancer cells. **a**. The phosphorylation of JNK and c-Jun were analyzed by western blotting at indicated concentration or treated with 4 μM of C-2 at indicated time points in BIU87 and EJ cells. **b** BIU87 and EJ cells were pretreated with 10 μM of SP600125 for 1 h and then treated with 4 μM of C-2 for an additional 24 h. The indicated proteins were detected by western blotting after immunoprecipitation with an antibody for Beclin-1. **c** The effects of JNK inhibitor SP600125 (10 μM) on 4 μM of C-2-induced LC3-II and p-JNK expression changes of BIU87 and EJ cells

To determine the signaling pathway that involved in C-2-induced autophagy, the effect of C-2 on JNK pathway was investigated. Results showed both JNK and c-Jun proteins been phosphorylated and activated, which was related to the up-regulation of p-JNK and p-c-Jun in C-2-treated BIU87 and EJ cells (Fig. 3a and Additional file 1: Figure S2). Moreover, whether C-2 led to dissociation of the Bcl-2/Beclin-1 and Bcl-xL/Beclin-1 complex through JNK pathway also was examined. Immunoprecipitation results revealed that Beclin-1 bound to Bcl-2 and Bcl-xL proteins in untreated cells, which was dissociated after C-2 treatment causing a decrease of Bcl-2/Bcl-xL expression. Interestingly, blocking JNK with pharmacological inhibitors SP600125 reversed the dissociation of the Bcl-2/Beclin-1 and Bcl-xL/Beclin-1 complex (Fig. 3b) and attenuated the expression level of LC3-II and Beclin1 proteins (Fig. 3c) caused by C-2 treatment, indicating that JNK pathway is a critical regulator of C-2-induced autophagy.

### Resisting C-2-induced apoptosis by p62 activated Nrf2 pathway in early time

**Fig. 4:**
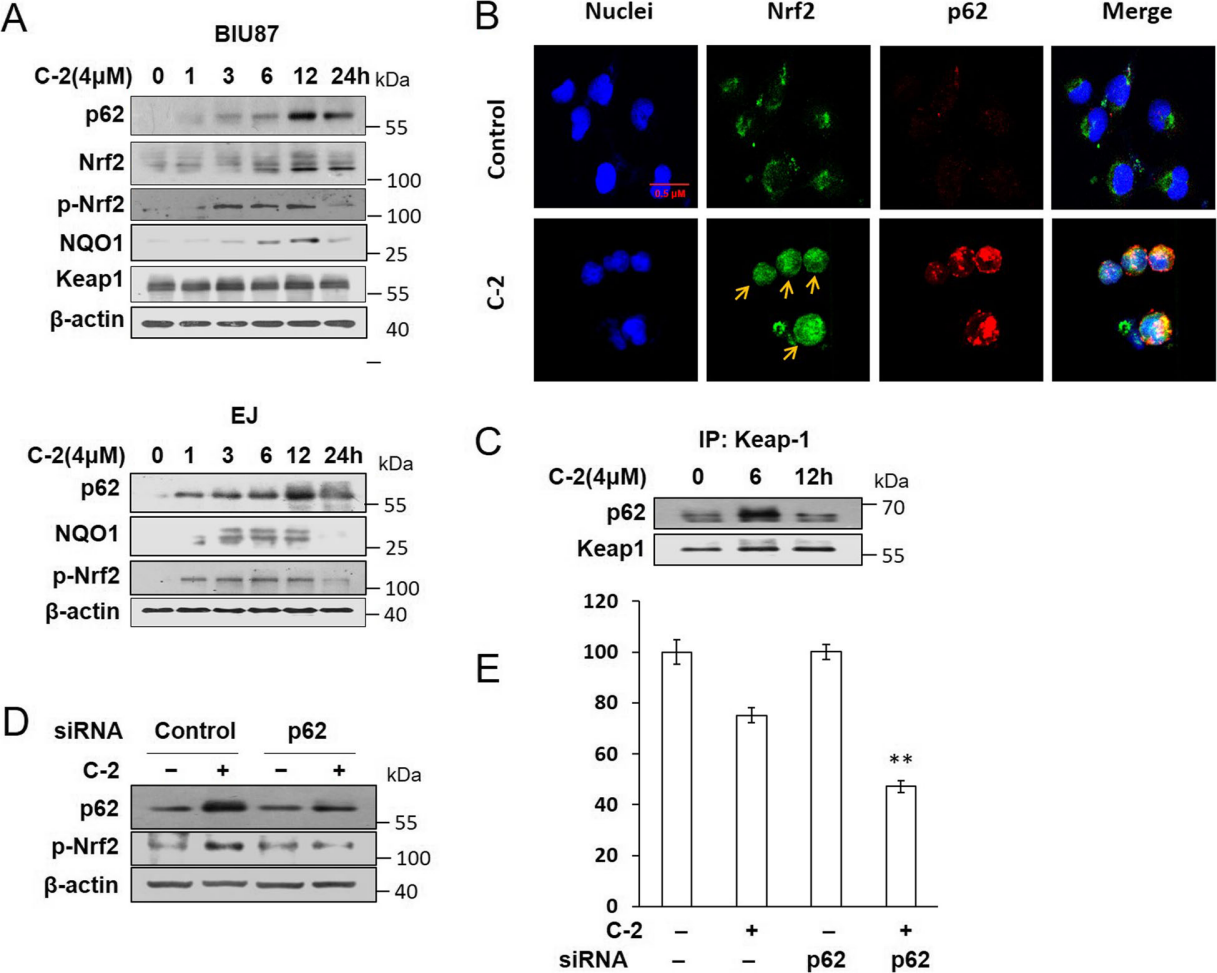
Resisting C-2 induced apoptosis by p62 activated Nrf2 pathway in early time. **a** Western blotting assay was used to detect the expression levels of p62, Nrf2, Keap1 and NQO1 after treated with 4 μM of C-2 for 24 h in BIU87 and EJ cells. **b** BIU87 cells were treated with C-2 for 6 h. The treated and untreated samples were stained with Nrf2 antibody (Green) and p62 antibody (Red) and DAPI (Blue) (magnification, 400X). The arrow was indicating Nrf2 nuclear translocation. **c** Immunoprecipitation assay showed the effect of C-2 on the binding of p62 and Keap1 proteins in BIU87 cells for 6 h and 12 h. **d** Western blotting assay showed the effect of p62 siRNA (20 nM) on expression of p62 and p-Nrf2 proteins in BIU87 cells. **e** MTT assay detected the effect of siRNA targeting to p62 on the survival rate of BIU87 cells incubated with 4 μM of C-2 for 6 h, **P < 0.02 vs C-2 treatment group

Since the p62 protein plays a significant role on autophagy process, the effect of p62 was investigated in our study. As shown in Fig. 4a, the expression levels of p62, p-Nrf2 and NQO1 was upregulated and reached its peak level at 12 h in C-2-treated bladder cancer cells, and the expression of Nrf2 and Keap1 has no obviously changes. In addition, we evaluated the effects of C-2 on target genes of Nrf2 such as NQO1, TrxR and IDH1, we found that C-2 clearly increases NQO1, TrxR and IDH1 mRNA levels (Additional file 1: Figure S3). Immunofluorescence assay showed accumulation of Nrf2 and p62 was higher within the nuclear following C-2 treatment (Fig. 4b). Immunoprecipitation noted that p62 bound with Keap1 at 6 h and then dramatically decreased (Fig. 4c), and utilization of p62 small interfering RNA (siRNA) decreased up-regulation of p-Nrf2 and the viability of BIU87 cells treated by C-2 (Fig. 4d and e), indicating that C-2 activated Nrf2 pathway through p62 to resisting apoptosis in early time.

### SP600125 block JNK-SQSTM1/p62-mediated Nrf2 anti-apoptotic pathway

**Fig. 5:**
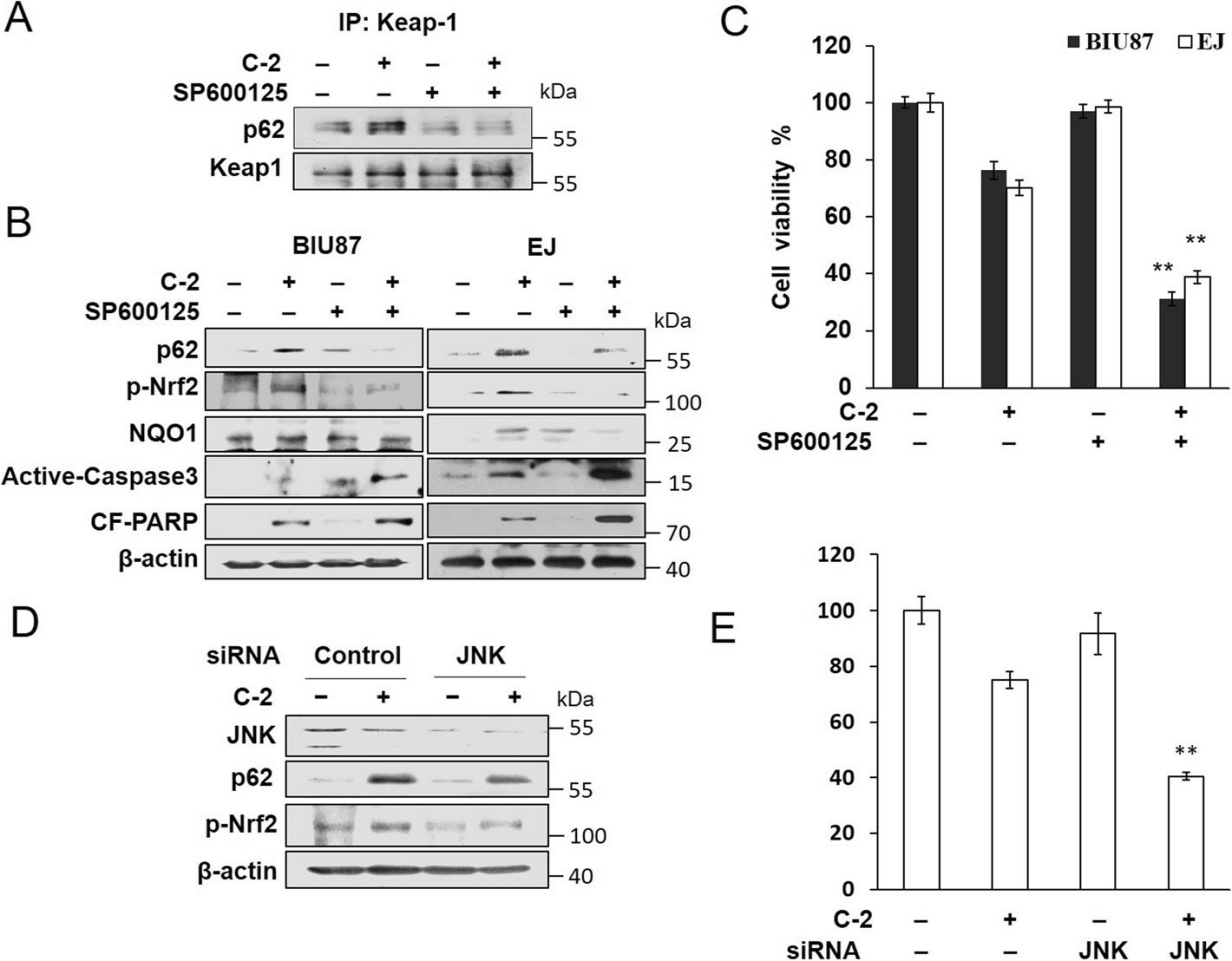
SP600125 block JNK-SQSTM1/p62-mediated Nrf2 anti-apoptotic pathway. Immunoprecipitation (**a**) and Western blotting (**b**) assay showed the effect of SP600125 (10 μM) on the expression changes of proteins in BIU87 cells incubated with 4 μM of C-2 for 6 h. (**c**) MTT assay demonstrated the effect of SP600125 (10 μM) on the cell viability of BIU87 and EJ treated by 4 μM of C-2 for 6 h. **P < 0.02 vs C-2 treatment group. (**d**) Western blotting assay showed the effect of JNK siRNA (20 nM) on expression of p62 and p-Nrf2 proteins in BIU87 cells. (**e**) MTT assay detected the effect of siRNA targeting to JNK on the survival rate of BIU87 cells incubated with 4 μM of C-2 for 6 h, **P < 0.02 vs C-2 treatment group

JNK inhibitor SP600125 was utilized to explore the potential effects of JNK on the p62-Keap1-Nrf2 pathway. As Fig. 5a, b and Additional file 1: Figure S4A showed that the binding of p62 and Keap1 proteins and the up-regulation of p62, p-Nrf2 and NQO1 caused by C-2 was attenuated by SP600125, whereas it enhanced the expression of CF-PARP and Active-caspase3 proteins, and the expression of Nrf2 has no obviously changes. MTT results showed SP600125 obviously increased the cell death of BIU87 and EJ caused by C-2 (Fig. 5c). In addition, JNK siRNA increased the cell death of BIU87 and decreased expression of p62 and p-Nrf2 caused by C-2, and the expression of Nrf2 has no obviously changes (Fig. 4d, e and Additional file 1: Figure S4B). These results suggested that blocking JNK resisted p62-Nrf2 anti-apoptotic pathway and lead to the switch from autophagy to apoptosis, and JNK is the key regulatory molecules of C-2 in bladder cancer cells.

### SP600125 potentiated the anti-tumor effect of C-2 in the xenograft nude mice model of EJ cells

**Fig. 6:**
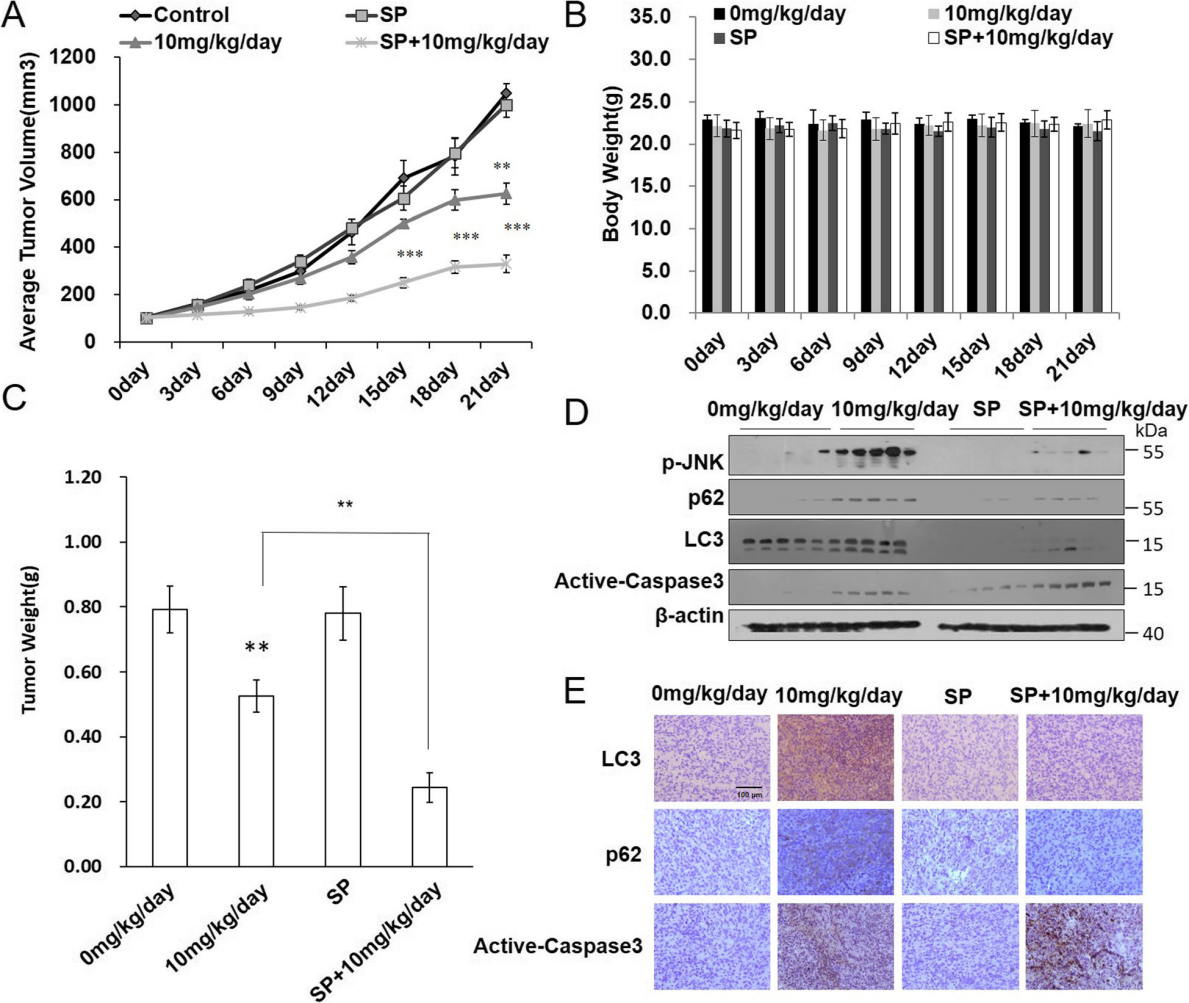
SP600125 potentiated the anti-tumor effect of C-2 in the xenograft nude mice model of EJ cells. Statistical analyses demonstrated that the average volume (**a**) and weight (**c**) of EJ xenografts tumor received C-2, SP600125 alone and in combination were significantly reduced. **P < 0.02, *** *P* < 0.01 vs Control group. **b** The body weight-time bar charts. Western blot assays (**d**) and immunohistochemical (**e**) were performed in xenograft tumors to demonstrate the expression changes of proteins induced by 10 mg/kg C-2 and C-2/SP600125 treatment (magnification 400X, scale bar 100 μm)

To evaluate the efficacy of C-2 treatment in vivo and to establish the mechanism of tumor growth inhibition in bladder cancer, EJ cells were implanted subcutaneously in nude mice. Since the establishment of the xenograft nude mouse tumor model with BIU87 cells is difficult, EJ bladder cancer cells were selected and study in vivo. SP600125 was utilized to further validate whether JNK pathway involved in C-2-induced autophagy in vivo*,* and the effect of JNK on tumor growth inhibition when SP600125 combined with C-2. Our results showed that C-2 treatment suppressed the growth of EJ tumors, and C-2/SP600125 group were significantly lower than those in mouse treated with vehicle or C-2 alone (Fig. 6a). There is no significant difference in mean body weights over time between vehicle control, C-2, SP600125 alone or C-2/SP600125 treated groups (Fig. 6b). The mean of wet tumor weights in C-2 treated mice was less than that of the control treated mice, and C-2/SP600125 exhibited more obviously effect than that of C-2 treated mice (Fig. 6c).

SP600125 was utilized to determine whether it potentiate the anti-tumor effect of C-2 through blockage of JNK-mediated autophagy, tumors collected after humanely sacrificing the mice were subjected to immunohistochemical and western blot analysis. As shown in Fig. 6d and e, in agreement with our in vitro observations, our in vivo results demonstrated SP600125 increased expression of active-Caspase3 and decreased the up-regulation of LC3 and p62 proteins induced by C-2 through western blot and immunohistochemistry analysis respectively in tumors. These results demonstrated SP600125 further enhanced the anti-tumor activity of C-2 in the xenograft nude mice model of EJ cells through attenuating autophagy.

## Discussion

Most patients with bladder cancer can be treated with organ-sparing therapy, radiotherapy and chemotherapy. Among these treatment schemes, chemotherapy is used as a primary or adjuvant therapy for treating cancer patients [1]. In recent years, naturally occurring botanicals and their derivatives are attracting considerable attention as cancer chemopreventive agents [17, 18]. Jaspine B, a natural product derived from a marine sponge *Pachastrissa sp.,* has exhibited potential antitumor activity in several tumor cell lines. However, detailed mechanism research of Jaspine B and its derivatives is still scarce, the role of autophagy in cell death and its cross-reaction with apoptosis are also still under investigation. Our previous study reported that Jaspine B derivatives compound 7f was discovered as an autophagy inducer and showed the best overall cytotoxicity on PC-3 cells [3]. In this study, another compound 7 g named C-2 exhibited strong cytotoxic effect against bladder cancer cells, and the underlying mechanism of autophagy and apoptosis triggered by C-2 and the crosstalk between autophagy and apoptosis were investigated in BIU87 and EJ cells.

It was reported that Jaspine B induced apoptosis in melanoma cells by interfering with ceramide metabolism [19]. In this study, the effect of apoptosis on bladder cancer cells induced by C-2 was also identified. Results showed that Bcl-2 family proteins alterations and mitochondrial membrane potential decreased by C-2 treatment in bladder cancer cells, indicating that mitochondria pathway involved in C-2 induced apoptosis. More importantly, evidence demonstrated that damage and subsequent dysfunction of mitochondria can lead to a wide range of disorders due to the impact on cellular metabolism and the release of the apoptotic factors [20]. Consequently, autophagy degrades damaged mitochondria is critical for the overall health of the cell, suggesting that a mitochondrial balance is involved in regulating both types of cell death: autophagy and apoptosis [21].

In addition to apoptosis, another process has a critical role in cancer therapy, which is autophagy. Myc is an oncogene that is often dysregulated in many cancers, and depletion of c-Myc impairs autophagy flux, thereby reducing phosphorylation of JNK and Bcl2. Knockdown of this oncogene transcription factor disrupts the formation of autophagosomes [22–25]. Autophagy is an extremely complex process including many signaling pathways, such as the target of rapamycin, phosphatidylinositon3-kinae-I/protein kinase B, guanosine triphosphate phosphohydrolase and MAPK pathways [26, 27]. Although it was reported that Jaspine B led to autophagy and anti-tumor activity in A549 cells [28], however, the mechanism of this action is not completely understood. In our study, the autophagy process induced by C-2 is associated with autophagy factors such as ATG7, ATG5, ATG3, p62, LC3 and Beclin-1, therefore, the ability of C-2 to induce autophagy in bladder cancer cell lines was confirmed. The autophagy flux covers autophagosome formation, maturation, fusion with lysosomes, subsequent breakdown and the release of macromolecules back into the cytosol. Here, Baf-A1 increased the accumulation of LC3-II proteins. In contrast, treatment with LY294002 reduced LC3-II accumulation in BIU87 cells, demonstrating that C-2 increased the autophagic flux [16]. And then went on to investigate the signaling pathways required for C-2-induced autophagy. Lin et al. [29] reported that cell autophagy through the AKT signaling pathway in T24 cells. Numerous published studies reported the activation of JNK had critical functions at upstream of autophagy induced by the deficiency of growth factors and nutrients, and stressing conditions [30, 31]. In this study, JNK activation triggered autophagy of bladder cancer cell through dissociation of the Bcl-2/Bcl-xL–Beclin-1 complex following C-2 treatment.

The p62, a selective substrate for autophagy, serves as a scaffold in autophagosomes through several structural domains, including the PB1 (Phox/Bem 1p), TB (TRAF6-binding), LIR (LC3-interacting region) and UBA (ubiquitin-associated) domains [32]. In this study, C-2 upregulated the expression of p62 and LC3-II at the protein levels. Similarly, tanshinone I, resveratrol and PMA increased autophagic flux with accumulation of intracellular p62 due to the enhancement of its synthesis as a compensation for autophagic degradation [33–35]. Thus, it can be assumed p62 induced by C-2 via its more synthesis compared with autophagic degradation.

Since p62 is also involved in various signal transduction pathways, such as the NF-κB pathway, Wnt signaling and apoptosis [36], furthermore, it is reported that p62 recruit RIPK1 to mediate necroptosis and lead to cell death occurs [37]. And p62 also contributed to Nrf2 signal pathway transduction to perform various functions. J. Shen et al. reported that p62-mediated Keap1/Nrf2 signaling pathway could induce IFNα antiviral response inhibits HCV replication [38], whereas Sun X et al. reported that activation of the p62-Keap1-Nrf2 pathway protects against ferroptosis in hepatocellular carcinoma cells [39]. In this study, p62 interacted with the Nrf2-binding site on Keap1 was investigated through immunoprecipitation, resulting in activation of Nrf2 pathway and transcriptional activation of Nrf2 target genes NQO1, then perform cellular defense mechanisms to resisting apoptosis effect in the early stage after C-2 treatment.

Next, we went on to investigate the effect of JNK signaling pathways on p62-Nrf2 axis and apoptosis induced by C-2, as well as the relation of them. Previous study reported that JNK pathways function could control the balance of autophagy and apoptosis in response to genotoxic stress [40]. Zhang et al. [41] reported inhibiting JNK activation could decrease DNA damage levels in human HepG2 hepatoma cells. However, numerous published studies reported that blockage of JNK-mediated autophagy pathway increased the anticancer activity in several cancer cells [42, 43]. In our study, SP600125 and utilization of JNK siRNA attenuated expression of p-Nrf2 and p62, promoted cytotoxicity and apoptosis triggered by C-2 in bladder cancer cells, moreover, knockdown of p62 by siRNA drastically enhanced the cell death effect of C-2. Taken together, blockage of JNK-p62-Nrf2 by SP600125 potentiates anti-cancer effect of C-2 through triggering the switch from autophagy to apoptosis induced by C-2.

In vivo study, we observed that 10 mg/kg C-2 significantly suppressed the growth of xenografts implanted EJ tumors. SP600125 combined with C-2 resulted in the increased efficacy of C-2 in vivo and further suppressed the growth of EJ tumor. Western blot and immunohistochemistry demonstrated SP600125 further enhanced the anti-tumor effect of C-2 through switching autophagy to apoptosis, consistent with our in vitro observations. More importantly, our study expects to provide new direction for design drugs or combination therapy.

In many stress pathways such as nutrient depletion, hypoxia or chemotherapeutic treatment, autophagy and apoptosis seem to occur simultaneously in most cells [4]. Autophagy, in special case, is an immediate response and can be pro-survival under conditions of cellular stress such as environmental stimulate or chemotherapy. However, basal levels of autophagy maintain cellular homeostasis but under stress conditions that high levels of autophagy and cell death have been observed, leading to the idea that autophagy may act as an executioner of cell death [44]. In this study, the pro-death role of autophagy induced by C-2 is however complicated due to the extensive crosstalk with apoptosis signaling pathways.

**Fig. 7:**
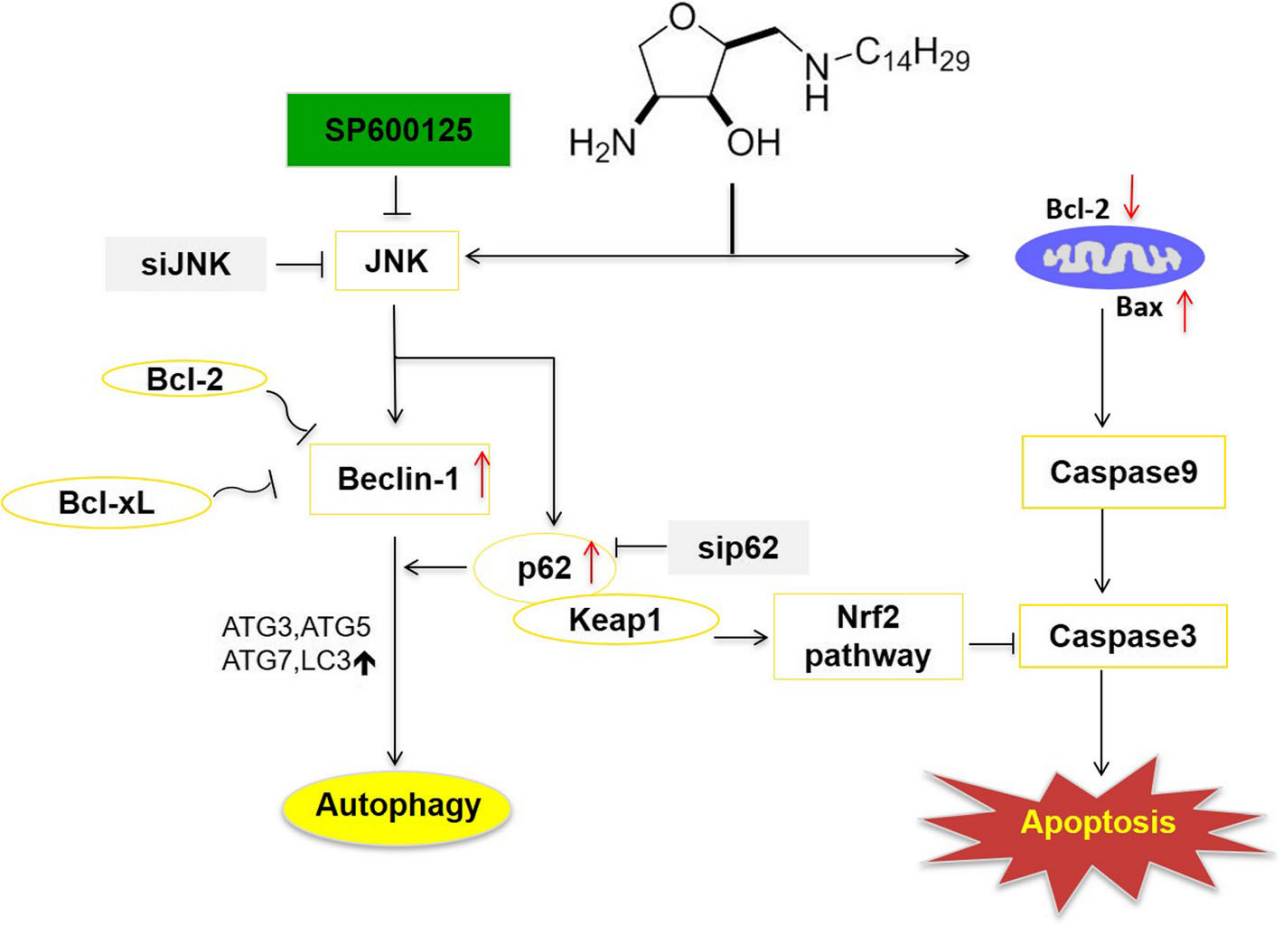
Summary of SP600125 enhances C-2-induced cell death in bladder cancer cells through resisting JNK- SQSTM1/p62- Nrf2 mediated anti-apoptotic pathway and switching autophagy to apoptosis

Overall, the mechanisms of the crosstalk between autophagy and apoptosis regulated by JNK-p62-Nrf2 axis in C-2-treated bladder cancer cells were confirmed (Fig. 7). To the best of our knowledge, C-2 as an autophagy inducer not only suppresses tumor growth but also the effect of that can be enhanced by SP600125. However, the effect of autophagy induced by C-2 on the biological function of apoptotic nodes and signaling pathways on bladder cancer requires further research and it is significant to clarify the interrelationships between them.

## Conclusions

Taken all together, these results suggest that C-2 maybe an attractive candidate for development of anti-tumor drugs targeting bladder cancer, and expecting it provides research basis and theoretical support for new drugs development.

## Supplementary information

**Additional file 1: Figure S1.** C-2 significantly induced apoptosis in human bladder cancer cells. (A) A dose-dependent induction of apoptosis by C-2 was demonstrated through flow cytometric analysis of Annexin V/PI stain assay. (B) The protein levels of Caspase9 were determined by western blotting assay at indicated concentrations for 24 h. For A, data are shown as mean ± s.d. (*n* = 3); **P < 0.01; ***P < 0.001 compared with control (Student’s *t* test). **Figure S2.** C-2-induced autophagy is associated with JNK pathway. The total of JNK and c-Jun were analyzed by western blotting at indicated concentration or treated with 4 μM of C-2 at indicated time points in BIU87 and EJ cells. **Figure S3.** Resisting C-2 induced apoptosis by p62 activated Nrf2 pathway in early time. The mRNA levels of NQO1, TrxR and IDH1 in BIU87 cells were detected by quantitative RT-PCR. Data are shown as mean ± s.d. (*n* = 3); ***P < 0.001 compared with control (Student’s *t* test). **Figure S4.** SP600125 block JNK-SQSTM1/p62-mediated Nrf2 anti-apoptotic pathway. (**A**) Western blotting assay showed the effect of SP600125 (10 μM) on the expression changes of Nrf2 protein in BIU87 cells incubated with 4 μM of C-2 for 6 h. **(B)** Western blotting assay showed the effect of JNK siRNA (20 nM) on expression of Nrf2 protein in BIU87 cells.

## Data Availability

Supplemental figure and associated figure legends are provided in supplemental material and are available online with the paper.

## Abbreviations

]PARP: Poly (ADP-ribose) polymerase
Atgs: Autophagy-related genes
Baf-A1: Bafilomycin A1
JNK: c-Jun N-terminal kinase
Keap1: The Kelch-like ECH-associated protein 1
LC3II: Microtubule-associated protein 1A/1B-light chain 3II
MMP: Mitochondrial membrane potential
Nrf2: Nuclear factor erythroid 2-related factor 2
p62: SQSTM1 (sequestosome 1)/p62
