# Supplementary material for: SP600125 enhances C-2-induced cell death by the switch from autophagy to apoptosis in bladder cancer cells
Source: J Exp Clin Cancer Res. 2019 Nov 4;38:448. doi: 10.1186/s13046-019-1467-6 (PMC6829950; doi:10.1186/s13046-019-1467-6)

**Supplementary materials**

**For**

**SP600125 enhances C-2-induced cell death by the switch from autophagy to apoptosis in bladder cancer cells**

Haiyang Yu, Chun-Li Wu, Xiangyu Wang, Qianhong Ban, Chunhua Quan, Mengbo Liu, Hangqi Dong, Jinfeng Li, Gi-Young Kim, Yung Hyun Choi, Zhenya Wang, Cheng-Yun Jin

**Figure Legend for Supplementary Data**

**Supplementary Figure 1. C-2 significantly induced apoptosis in human bladder cancer cells.** (**A**) A dose-dependent induction of apoptosis by C-2 was demonstrated through flow cytometric analysis of Annexin V/PI stain assay. (**B**) The protein levels of Caspase9 were determined by western blotting assay at indicated concentrations for 24 h. For **A**, data are shown as mean ± s.d. (n = 3); ***P* < 0.01; ****P* < 0.001 compared with control (Student’s *t* test).

**Supplementary Figure 2. C-2-induced autophagy is associated with JNK pathway.** The total of JNK and c-Jun were analyzed by western blotting at indicated concentration or treated with 4 μM of C-2 at indicated time points in BIU87 and EJ cells.

**Supplementary Figure 3. Resisting C-2 induced apoptosis by p62 activated Nrf2 pathway in early time.** The mRNA levels of NQO1, TrxR and IDH1 in BIU87 cells were detected by quantitative RT-PCR. Data are shown as mean ± s.d. (n = 3); ****P* < 0.001 compared with control (Student’s *t* test).

**Supplementary Figure 4. SP600125 block JNK-SQSTM1/p62-mediated Nrf2 anti-apoptotic pathway.** (**A**) Western blotting assay showed the effect of SP600125 (10 μM) on the expression changes of Nrf2 protein in BIU87 cells incubated with 4 μM of C-2 for 6 h. **(B)** Western blotting assay showed the effect of JNK siRNA (20 nM) on expression of Nrf2 protein in BIU87 cells.

**Supplementary Figure 1**


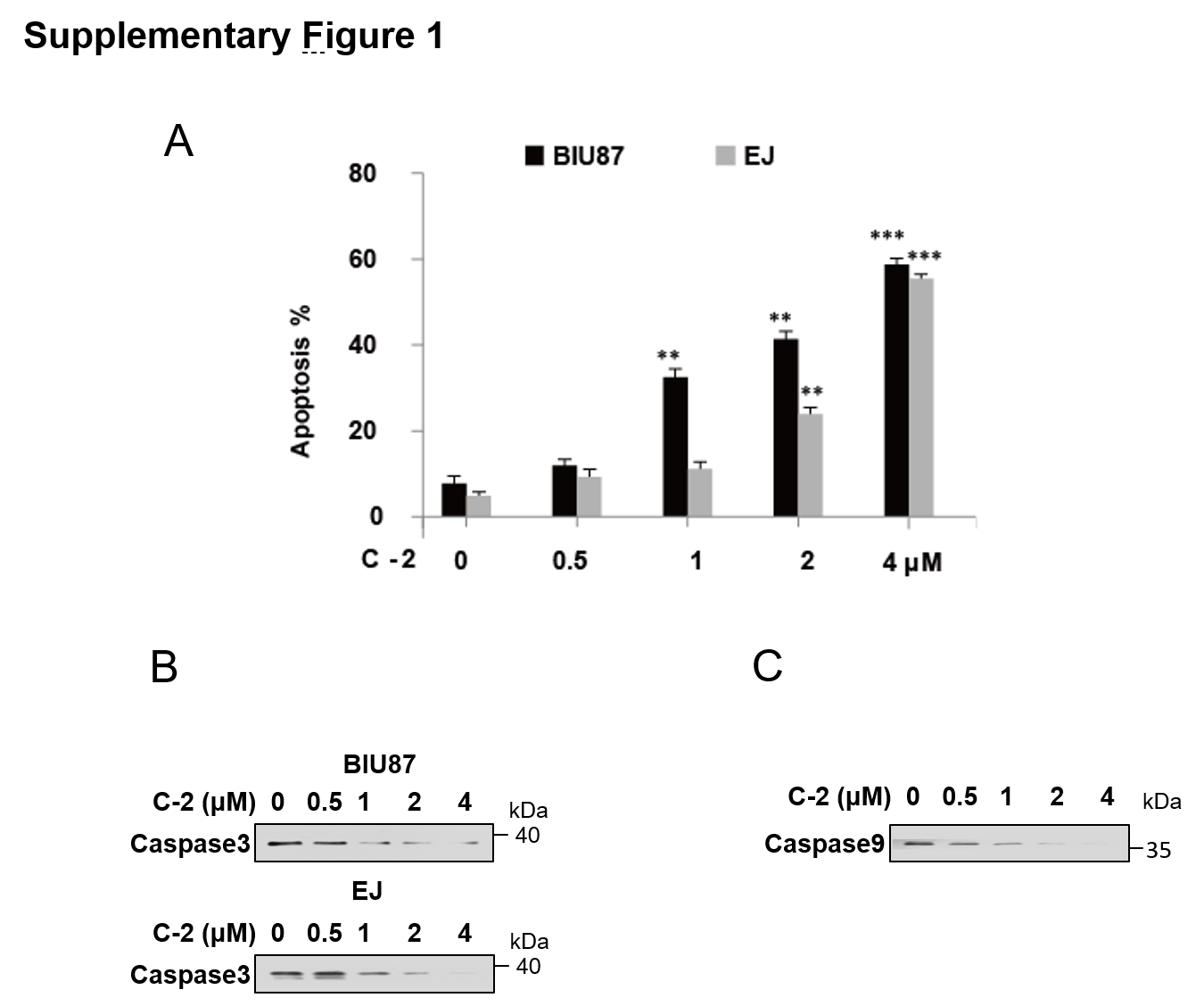


**Supplementary Figure 2**


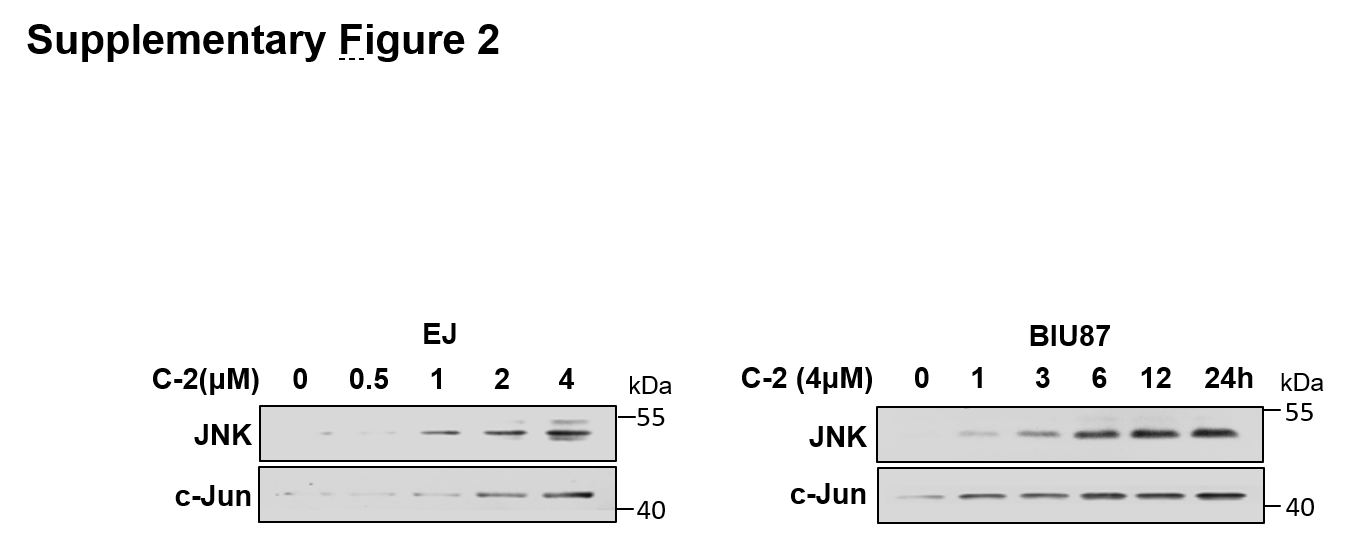


**Supplementary Figure 3**


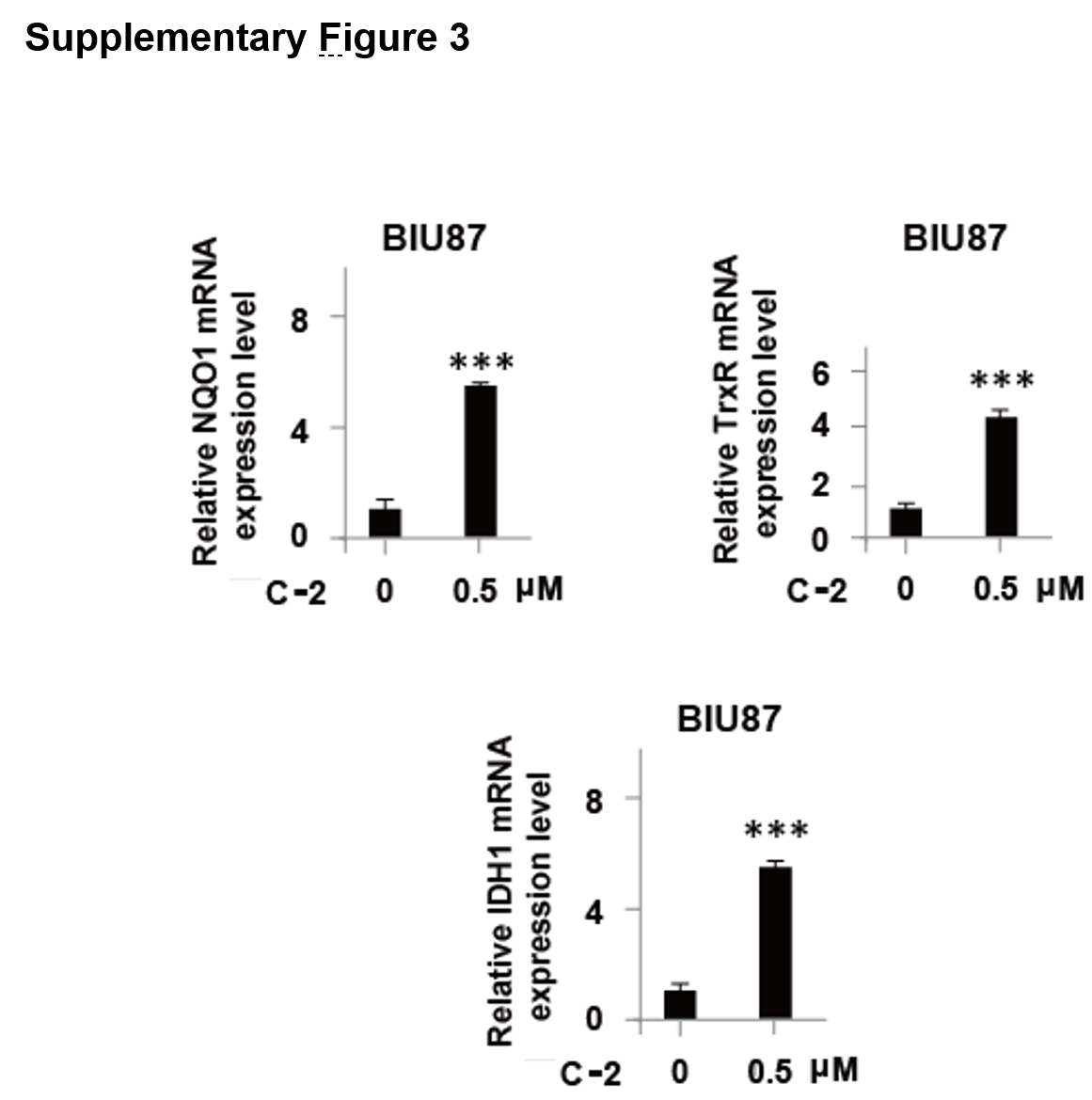


**Supplementary Figure 4**


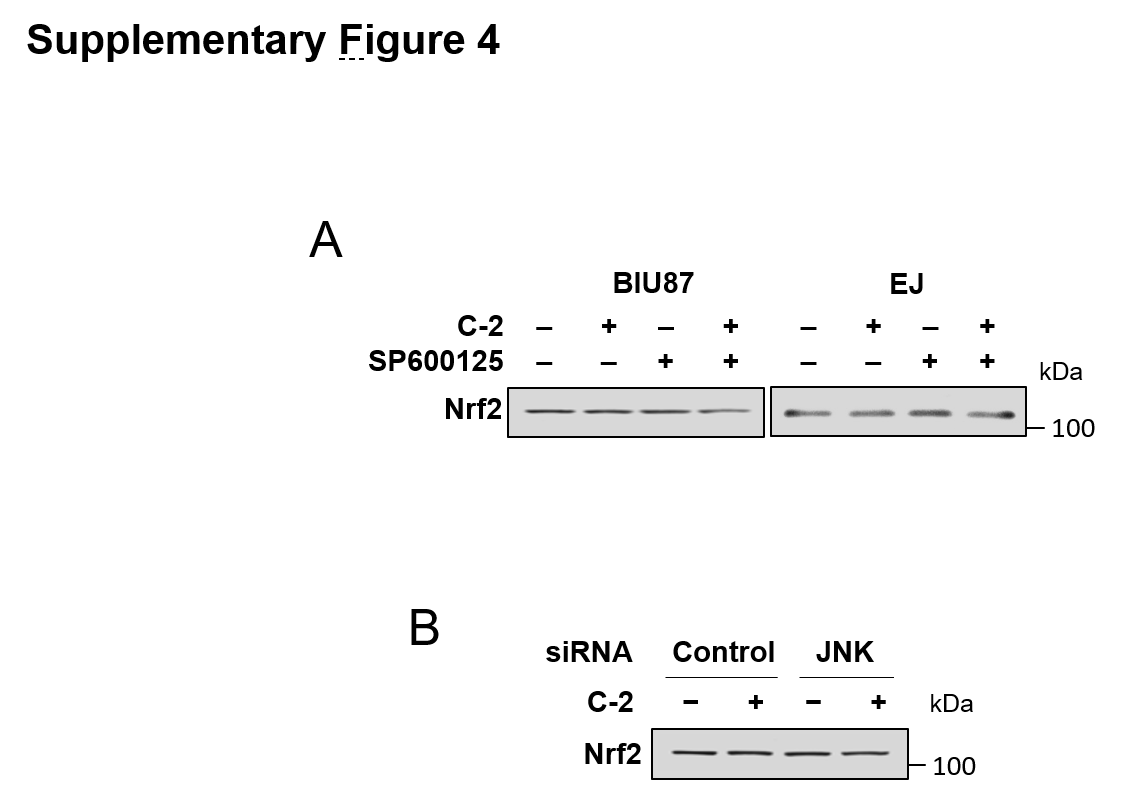

Supplement: Supplementary file 1 — Additional file 1: Figure S1. C-2 significantly induced apoptosis in human bladder cancer cells. (A) A dose-dependent induction of apoptosis by C-2 was demonstrated through flow cytometric analysis of Annexin V/PI stain assay. (B) The protein levels of Caspase9 were determined by western blotting assay at indicated concentrations for 24 h. For A, data are shown as mean ± s.d. (n = 3); **P < 0.01; ***P < 0.001 compared with control (Student’s t test). Figure S2. C-2-induced autophagy is associated with JNK pathway. The total of JNK and c-Jun were analyzed by western blotting at indicated concentration or treated with 4 μM of C-2 at indicated time points in BIU87 and EJ cells. Figure S3. Resisting C-2 induced apoptosis by p62 activated Nrf2 pathway in early time. The mRNA levels of NQO1, TrxR and IDH1 in BIU87 cells were detected by quantitative RT-PCR. Data are shown as mean ± s.d. (n = 3); ***P < 0.001 compared with control (Student’s t test). Figure S4. SP600125 block JNK-SQSTM1/p62-mediated Nrf2 anti-apoptotic pathway. (A) Western blotting assay showed the effect of SP600125 (10 μM) on the expression changes of Nrf2 protein in BIU87 cells incubated with 4 μM of C-2 for 6 h. (B) Western blotting assay showed the effect of JNK siRNA (20 nM) on expression of Nrf2 protein in BIU87 cells. [file 13046_2019_1467_MOESM1_ESM.docx]
